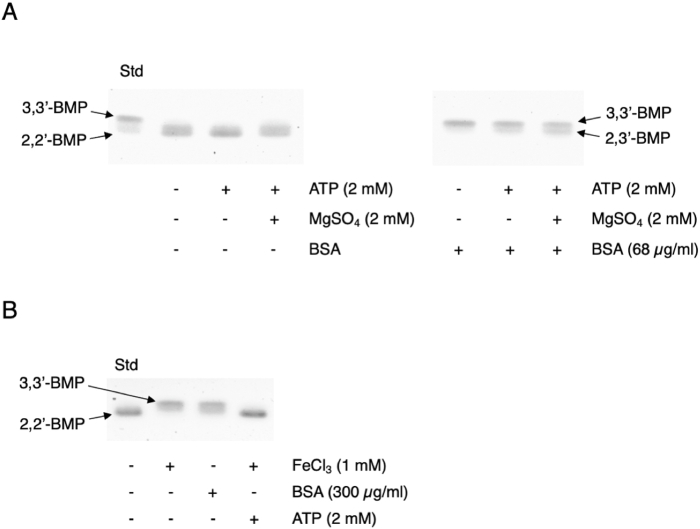# Isomerization of bis(monoacylglycero)phosphate by acyl migration

**DOI:** 10.1016/j.jlr.2025.100789

**Published:** 2025-03-29

**Authors:** Akira Abe, Vania Hinkovska-Galcheva, Rakesh Verma, James A. Shayman

**Affiliations:** Department of Internal Medicine, University of Michigan, Ann Arbor, MI, USA

**Keywords:** albumin, bis(monoacylglycero)phosphate, ferroptosis, lysosomal phospholipase A2, PLA2G15

## Abstract

Bis(monoacylglycero)phosphates (BMPs) are biologically functional acidic lipids present in late endosomes and lysosomes. We recently reported that lysosomal phospholipase A2 (LPLA2, PLA2G15), the lysosomal enzyme mediating BMP catabolism, degrades BMP isomers with distinct substrate specificity. Specifically, *sn*-(3-oleoyl-2-hydroxy)-glycerol-1-phospho-*sn*-1’-(3′-oleoyl-2′-hydroxy)-glycerol (S,S-(3,3′-diC_18:1_)-BMP) is a significantly better substrate for LPLA2 than S,S-(2,2′-diC_18:1_)-BMP. S,S-(2,2′-diC_18:1_)-BMP is generally considered the only biologically relevant BMP isomer. We investigated the isomerization of S,S-(2,2′-diC_18:1_)-BMP to (S,S-(3,3′-diC_18:1_)-BMP) in vitro and in cells. Thin-layer chromatography was used to distinguish S,S-(3,3′-diC_18:1_)-BMP from S,S-(2,2′-diC_18:1_)-BMP. S,S-(2,2′-diC_18:1_)-BMP/1,2-di-*O*-(9Z-octadecenyl)-*sn*-glycero-3-phosphocholine liposomes were incubated at varying pH in the presence or absence of test substances. First, we studied bovine serum albumin, which is known to promote isomerization of 1-acyl-2-lysophosphatidylcholine. The formation of S,S-(3,3′-diC_18:1_)-BMP in the presence of albumin increased in a time-dependent and albumin concentration-dependent manner under neutral conditions and was dependent on pH and the molar ratio of S,S-(2,2′-diC_18:1_)-BMP in liposomes. Treatment of isomeric products generated during isomerization reaction with *sn*-1,3-specific lipase produced both oleic acid but also lyso-phosphatidylglycerol, indicating that the conversion of S,S-(2,2′-diC_18:1_)-BMP to S,S-(3,3′-diC_18:1_)-BMP is preceded via S,S-(2,3′-diC_18:1_)-BMP. S,S-(3,3′-diC_18:1_)-BMP formed was preferentially degraded by LPLA2 over the S,S-(2,2′-diC_18:1_)-BMP. Proteins such as HSP70 and human serum albumin and metal ions such as Fe^3+^ and Zn^2+^ acted as cofactors promoting the isomerization of S,S-(2,2′-diC_18:1_)-BMP under neutral conditions. At baseline, RAW 264.7 cells showed nonnegligible amounts of *sn*-1,3-specific lipase-sensitive BMPs. However, lipase-sensitive BMPs were increased by exposure to chloroquine or NH_4_Cl, suggesting that cells undergo S,S-(2,2′-diacyl)-BMP isomerization upon alkalinization of intracellular acidic compartments.

Bis(monoacylglycero)phosphate (BMP) is a minor cellular lipid component in most eukaryote cells and tissues, comprising less than 1% of the cellular phospholipid. An exception is found in alveolar macrophages where BMP accounts more than 10% of total phospholipid (1). BMPs are characterized by an unusual *sn*-1:*sn*-1′ stereo-configuration (2) and are abundant in late endosomes and lysosomes where BMPs comprise approximately 15% of the phospholipids found in these acidic compartments (3). The naturally occurring BMP isomer is generally thought to be *sn*-(2-monoacyl-3-hydroxy)-glycerol-1-phospho-*sn*-1’-(2′-monoacyl-3′-hydroxy)-glycerol (S,S-(2,2′-diacyl)-BMP). BMPs are implicated in many biological events including internal vesicle formation in the endosomes, the regulation of the trafficking and efflux of cholesterol associated with Niemann Pick 1 and 2 in lysosomes, the activation of lysosomal hydrolases such as acidic sphingomyelinase, lysosomal phospholipase A2 (LPLA2, PLA2G15), β-glucocerebrosidase, the stabilization of endosome/lysosomal membranes via binding with Hsp70, and autolysosome formation (4). Despite these potentially important functions, studies characterizing the pathways of BMP metabolism have not been thoroughly studied.

It has been shown that phosphatidylglycerol (5) is converted to BMP through deacylation by phospholipase A2, followed by acylation at the *sn*-2′, rearrangement of the phosphoryl group and reacylation at the *sn*-2 (6, 7, 8). Recently, it was reported that CLN5 is the BMP synthase (9), but the authors did not demonstrate that S,S-(2,2′-diacyl)-BMP isomer was the specific product of this CLN5 activity, and this finding was not confirmed in a subsequent publication (10). PLD3 and PLD4 have been identified as lipases specifically required for S,S-(2,2′-diacyl)-BMP synthesis (11). These enzymes were shown to catalyze a stereo-conversion of R,S-phosphatidylglycerol to S,S-(3,3′-diacyl)-BMP. However, the resultant S,S-(3,3′-diacyl)-BMP requires deacylation at *sn*-3 and *sn*-3′ and reacylation at *sn*-2 and *sn*-2′ to form the native S,S’-(2,2′-diacyl)-BMP isomer. Prior work has reported the catabolism of BMP by an acid phosphodiesterase (12) and the enzyme alpha/beta hydrolase domain containing 6 (ABHD6) (13).

LPLA2, originally characterized a 1-*O*-acylceramide synthase, is a lysosomal and late endosomal enzyme that maintains general glycerophospholipid homeostasis in cells. Alveolar macrophages obtained from bronchoalveolar lavage of LPLA2-deficient mice are characterized by higher levels of glycerophospholipids, including BMPs, than those obtained from WT mice (14, 15). The accumulation of BMPs in LPLA2-deficient mice alveolar macrophages is reversed by the administration of active recombinant LPLA2 (15).

LPLA2 catabolism of glycerophospholipids is inhibited by cationic amphiphilic drugs such as amiodarone (AMD). The proposed mechanism of inhibition is through the interruption of the electrostatic interaction between the enzyme and phospholipid membranes (16). Our recent study showed that BMPs accumulated in macrophage RAW-264.7 cells due to AMD were effectively reduced by treatment with AMD-free culture medium containing enzymatically active recombinant LPLA2. However, the inactivation of LPLA2 by diisopropyl fluorophosphate failed to reverse the increase in BMP levels in AMD-treated RAW-264.7 cells. In the same study, we reported that LPLA2 degrades BMP isomers including the naturally occurring form S,S-(2,2′-diC_18:1_)-BMP with distinct substrate specificity (17). These results implicate LPLA2 with BMP catabolism in acidic cellular compartments.

Interestingly, 1 BMP isomer, S,S-(3,3′-diC_18:1_)-BMP, was found to be 10-fold more efficiently deacylated by LPLA2 compared to S,S-(2,2′-diC_18:1_)-BMP (17). S,S-(3,3′-diacyl)-BMP is thought to be thermodynamically more stable than S,S-(2,2′-diacyl)-BMP and to be formed by spontaneous acyl migration of S,S-(2,2′-diacyl)-BMPs. However, the acyl groups in natural BMP molecules in baby hamster kidney cells are mostly located at the *sn*-2 and sn-2′ positions (18). In addition, BMPs in the S,S-(2,2′-diacyl) configuration are biologically functional lipids (19). Our recent findings regarding substrate specificity of LPLA2 for BMPs raises the possibility that the intracellular isomerization of S,S-(2,2′-diacyl)-BMP to S,S-(3,3′-diacyl)-BMP regulates the catabolism of BMP by LPLA2 (17).

In this communication, we report that S,S-(2,2′-diacyl)-BMP is converted to S,S-(3,3′-diacyl)-BMP in the presence of factors such as bovine and human serum albumin (HSA), HSP70, or metal ions under neutral conditions, and that the S,S-(3,3′-diacyl)-BMP formed is much more efficiently degraded by LPLA2 than S,S-(2,2′-diacyl)-BMP in vitro. In addition, our current results indicate that RAW-264.7 cells contain nonnegligible amounts of *sn*-1,3-specific lipase-sensitive BMPs, but that such BMPs increase upon increasing lysosomal pH. These findings suggest the existence of an S,S-(2,2′-diacyl)-BMP isomerization pathway that precedes LPLA2-mediated BMP degradation.

## Materials and methods

### Reagents

*sn*-(3-Oleoyl-2-hydroxy)-glycero-1-phospho-*sn*-1’-(3′-oleoyl-2′-hydroxy)-glycerol (S,S-(3,3′-diC18:1)-BMP), 1,2-di-*O*-(9*Z*-octadecenyl)-*sn*-glycero-3-phosphocholine (DODPC), 1,2-dioleoylphosphatidylcholine (DOPC), sulfatide, and *N*-acetylsphingosine were obtained from Avanti Polar Lipids Corp. (Alabaster, AL). *sn*-(2-Oleoyl-3-hydroxy)-glycerol-1-phospho-*sn*-1'-(2′-oleoyl-3′-hydroxy)-glycerol (S,S-(2,2′-diC_18:1_)-BMP) was from Echelon (Salt Lake City, UT). Palmitic acid, oleic acid, diisopropyl fluorophosphate, and lipase from *Rhizomucor miehei* were from Sigma-Aldrich (St. Louis, MO). Recombinant human LPLA2 was from Proteos (Kalamazoo, MI), and high-performance TLC silica gel plates, 10 × 20 cm, were from Merck (Darmstadt, Germany). Recombinant human HSP70/HSA1A protein and progranulin were from Bio-techne (Minneapolis, MN). Advanced glycation end product-BSA was from Cayman (Ann Arbor, MI).

### Preparation of liposomes

Liposomes consisted of DODPC and S,S-(2,2′-diC_18:1_)-BMP or S,S-(3,3′-diC_18:1_)-BMP in the molar ratio of 7:3. DODPC in chloroform (C) and BMP in chloroform/methanol (C/M) (2:1, v/v) were delivered in a glass tube and dried down under a stream of nitrogen (N_2_) gas. The dried lipids were dispersed in 0.15 M sodium chloride by a probe-type sonicator for 8 min while cooling in an ice-water bath.

### Detection of conversion of S,S-(2,2′-dioleoyl)-BMP to S,S-(3,3′-dioleoyl)-BMP

The reaction mixture contained liposomes (7.2 μM as phospholipid) consisting of DODPC/S,S-(2,2′-diC_18:1_)-BMP or DOPC/S,S-(3,3′-diC_18:1_)-BMP (molar ratio 7:3), 50 mM sodium chloride, 15.7 mM sodium acetate (pH 4.5), or 15.7 mM Hepes (pH 7.4) in the presence or absence of 667 μg/ml BSA. The reaction was initiated by adding BSA, kept for 4 h at 37°C and terminated by mixing with C/M. The ratio of C/M/aqueous solution was 2:1:0.8, v/v. The resultant organic phase obtained after centrifugation at 800 *g* for 5 min at room temperature was transferred to another glass tube, dried down under a stream of N_2_ gas, and applied to an high-performance TLC plate. The plate was developed in a solvent system consisting of C/M/acetone/acetic acid/water (45:15:10:10:2, v/v). To identify the reaction products, S,S-(2,2′-diC_18:1_)-BMP and S,S-(3,3′-diC_18:1_)-BMP prepared from each purchased BMP reagent were used as standards. The plate was dried with a hair drier and immersed in 8% (w/v) CuSO_4_•5H_2_O, 6.8% (v/v) H_3_PO_4_, 32% (v/v) methanol. The uniformly wet plate was again briefly dried with a hair dryer and charred for 15 min in a 150°C oven. The stained products were scanned with a CanoScan 9000F Mark II and analyzed by ImageJ.

In the time dependence study of the conversion of S,S-(2,2′-diC_18:1_)-BMP to S,S-(3,3′-diC_18:1_)-BMP, the reaction mixture was incubated for 0, 0.5, 1, 2, and 4 h at 37°C at pH 7.4. In the BSA concentration dependence study for the conversion, the reaction mixture containing different concentrations of BSA (0.00, 33.3, 100, 333, 667, and 1,000 μg/ml) was incubated for 4 h at 37°C at pH 7.4.

### Degradation of tentative S,S-(3,3′-dioleoyl)-BMP by sn-1,3-specific lipase

The reaction mixtures that contained liposomes (7.2 μM as phospholipid) consisting of DODPC/S,S-(3,3′-diC_18:1_)-BMP (molar ratio 7:3), 50 mM sodium chloride, and 15.7 mM Hepes (pH 7.4) in the absence or presence of 667 μg/ml BSA were incubated for 4 h at 37°C. In addition, those reaction mixtures were treated with or without 0.42 U/ml *sn*-1,3-specific lipase from *R. miehei* for 1 h at 37°C. The resultant products were extracted and detected as described above.

### Degradation of S,S-(3,3′-dioleoyl)-BMP formed from S,S-(2,2′-dioleoyl)-BMP by hLPLA2

Reaction mixtures that contained liposomes (7.2 μM as phospholipid) consisting of DODPC/S,S-(2,2′-diC_18:1_)-BMP (molar ratio 7:3), 50 mM sodium chloride, and 15.7 mM Hepes (pH 7.4) in the absence or presence of 667 μg/ml BSA were incubated for 4 h at 37°C. After incubation, the pH of those reaction mixtures was adjusted to 4.5 using 2.08 M sodium acetate (pH 4.5) (final concentration of sodium acetate: 54 mM), and then the pH-adjusted reaction mixtures were treated with or without 3.5 μg/ml hLPLA2 for 1 h at 37°C. The resultant products were extracted and detected as described above.

### Lipase-sensitive BMP in RAW-264.7 cells

RAW-264.7 macrophage-like cells were seeded into 35 mm diameter dishes (3 × 10^6^ cells per dish) in DMEM containing 10% FCS, 2 mM L-glutamine, 100 U/ml penicillin, and 100 mg/ml streptomycin (basal culture conditions) and incubated at 37°C in a humidified atmosphere with 5% CO_2_ overnight. The cells were treated with or without 20 μM chloroquine (CHL) or 20 mM NH_4_Cl for 24 or 48 h in the presence of FCS. After treatment, the cells were washed three times with PBS, scraped into 0.25 M sucrose buffer containing 10 mM Hepes (pH 7.4) and 1 mM EDTA, and then subjected to centrifugation at 350 *g*. The cell pellet was dispersed in the same sucrose buffer and briefly sonicated to prepare cell homogenates. The lipid extracts prepared from the cell homogenates (90 μg of protein) were incubated with or without *sn*-1,3-specific lipase from *R. miehei* (0.84 U/ml) for 1 h at 37°C. The reaction mixture contained 135 mM NaCl and 20 mM Hepes (pH 7.4). After incubation, the lipids were extracted and detected as described above.

## Results

### Isomerization of S,S-(2,2′-dioleoyl)-BMP to S,S-(3,3′-dioleoyl)-BMP

**Fig. 1:**
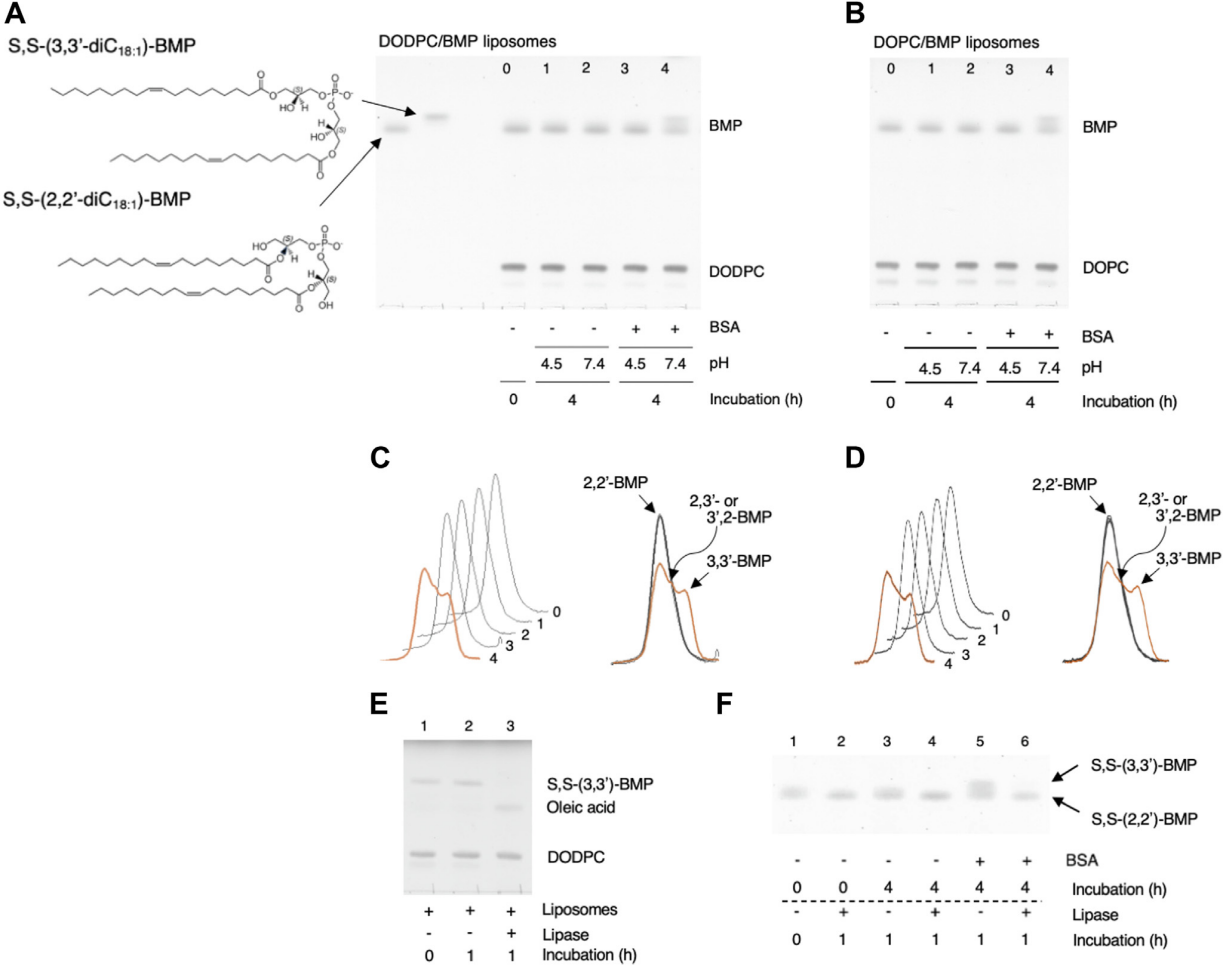
Conversion of S,S-(2,2′-dioleoyl)-BMP to S,S-(3,3′-dioleoyl)-BMP in the presence of BSA under neutral conditions. Representative TLC showing the conversion of DODPC/S,S-(2,2′-diC_18:1_)-BMP to DODPC/S,S-(3,3′-diC_18:1_)-BMP in DODPC/BMP liposomes (A) and DOPC/BMP liposomes (B), and representative TLC scan profiles of panel A and panel B (C and D, respectively). The reaction mixture containing DODPC/S,S-(2,2′-diC_18:1_)-BMP or DOPC/S,S-(2,2′-diC_18:1_)-BMP liposomes (molar ratio 7:3, 7.2 μM as phospholipid) was incubated at pH 4.5 or 7.4 in the presence or absence of 0.667 mg/ml BSA for 4 h at 37°C. The resultant reaction products were applied to an HPTLC plate. The plate was developed in a solvent system consisting of C/M/acetone/acetic acid/water (45:15:10:10:2, v/v) (A and B). Individual lanes (0–4) of each plate were scanned (panels C and D, left) and the resultant curves were superimposed (panels C and D, right). Representative TLCs showing the sensitivity of S,S-(2,2′-diC_18:1_)-BMP and S,S-(3,3′-diC_18:1_)-BMP to *sn*-1,3-specific lipases (E and F). In panel E, the reaction mixtures containing DODPC/S,S-(2,2′-diC_18:1_)-BMP liposomes were treated at pH 7.4 with or without 0.42 U/ml *sn*-1,3-specific lipase from *Rhizomucor miehei* for 1 h at 37°C. In panel F, the reaction mixture containing DODPC/S,S-(2,2′-diC_18:1_)-BMP liposomes incubated at pH 7.4 in the absence or presence of 0.667 mg/ml BSA for 4 h at 37°C. In addition, these reaction mixtures were treated with or without *sn*-1,3-specific lipase from *R. miehei* for 1 h at 37°C. The plates of panel E and F were developed in solvent systems consisting of C/M/28% ammonium solution (65:35:5, v/v) and C/M/acetone/acetic acid/water (45:15:10:10:2, v/v), respectively. For details, see the Materials and methods. 2,2′-BMP and 3,3′-BMP indicate *sn*-(2-oleoyl-3-hydroxy)-glycerol-1-phospho-*sn*-1′-(2′-oleoyl-3′-hydroxy)-glycerol (S,S-(2,2′-diC_18:1_)-BMP) and *sn*-(3-oleoyl-2-hydroxy)-glycerol-1-phospho-*sn*-1′-(3′-oleoyl-2′-hydroxy)-glycerol (S,S-(3,3′-diC_18:1_)-BMP), respectively. BMP, bis(monoacylglycero)phosphate; C/M, chloroform/methanol; DODPC, 1,2-di-*O*-(9Z-octadecenyl)-*sn*-glycero-3-phosphocholine; HPTLC, high-performance thin layer chromatography.

The stability of S,S-(2,2′,diC_18:1_)-BMP incorporated in DODPC-based liposomes under acidic and neutral conditions was first confirmed. DODPC is a dialkenyl-glycerophospholipid and is not hydrolyzed by any lipase or phospholipase. With the TLC system employed the mobility of S,S-(2,2′-diC_18:1_)-BMP was slower than that of S,S-(3,3′-diC_18:1_)-BMP and distinguishable from S,S-(3,3′-diC_18:1_)-BMP (Fig. 1A, left two lanes). We chose to use TLC as a convenient characterization method to distinguish between S,S-(2,2′,diC_18:1_)-BMP and S,S-(3,3′,diC_18:1_)-BMP.

To assess the stability of S,S-(2,2′-diC_18:1_)-BMP, DODPC/S,S-(2,2′-diC_18:1_)-BMP liposomes dispersed in sodium acetate (pH 4.5) or Hepes (pH 7.4) were incubated at 37°C for 4 h. The resultant reaction solutions were fractionated using C/M and the organic layer was subjected to TLC analysis. As shown in Fig. 1A, C (lanes 0, 1 and 2), the content of S,S-(2,2′-diC_18:1_)-BMP was unchanged by 4 h incubation at 37°C under both acidic and neutral conditions. In addition, it was little changed even after 22 h incubation (data not shown). These results indicate that unlike 2-acyl-lysoPC, S,S-(2,2′-diC_18:1_)-BMP incorporated into phospholipid liposomes does not undergo spontaneous acyl migration.

It has been reported that the acyl migration of the acyl group at the *sn*-2 position of 2-acyl-lysoPC to the *sn*-1 position is significantly enhanced by BSA (20, 21, 22). Based on structural relationships, we considered whether the acyl migration mechanism of 2-acyl-lysoPC could be similar for S,S-(2,2′-diC_18:1_)-BMP. When the liposomes were treated for 4 h at 37°C in the presence of BSA at either pH 4.5 or 7.4, the content of S,S-(2,2′-diC_18:1_)-BMP treated at neutral pH was reduced and a faster migrating band appeared, but not at acidic pH (Fig. 1A, C, lanes 3 and 4). This new band showed a similar mobility to that of commercial S,S-(3,3′-diC_18:1_)-BMP (Fig. 1A, C, lane 4). The same change was also observed in DOPC/S,S-(2,2′,diC_18:1_)-BMP liposomes (Fig. 1B, D). In both studies, no significant amounts of oleic acid or lyso-phosphatidylglycerol (lyso-PG) were released.

S,S-(3,3′-diC_18:1)_-BMP incorporated in DODPC liposomes was completely degraded by *sn*-1,3-specific lipase of *R. miehei*, which deacylates fatty acids conjugated to the primary alcohol group of the glycerol moiety, and oleic acid release was observed (Fig. 1E, lane 3). In contrast, S,S-(2,2′-diC_18:1_)-BMP in DODPC liposomes was resistant to the same enzyme (Fig. 1F, lanes 2 and 4). In addition, S,S-(3,3′-diC_18:1_)-like BMP converted from S,S-(2,2′-diC_18:1_)-BMP in the presence of BSA at pH 7.4 (Fig. 1F, lane 5) was degraded by *sn*-1,3-specific lipase (Fig. 1F, lane 6), but the S,S-(2,2′-diC_18:1_)-BMP remained in the same reaction mixture was unchanged (Fig. 1F, lane 6). These results indicate that the BMP formed in the presence of BSA was in fact S,S-(3,3′-diC_18:1_)-BMP.

In addition, the stained bands (Fig. 1A–D, spectra 4) observed between S,S-(2,2′-diC_18:1_)-BMP and S,S-(3,3′-diC_18:1_)-BMP bands, predicted to originate from S,S-(2,3′-diC_18:1_)-BMP or S,S-(3,2′-diC_18:1_)-BMP, were also sensitive to the *sn*-1,3-specific lipase (Fig. 1F, lanes 5 and 6), consistent with the presence of S,S-(2,3′-diC_18:1_)-BMP or S,S-(3,2′-diC_18:1_)-BMP.

### Dependence of S,S-(2,2′,diC_18:1_)-BMP isomerization on time, albumin concentration, and pH

**Fig. 2:**
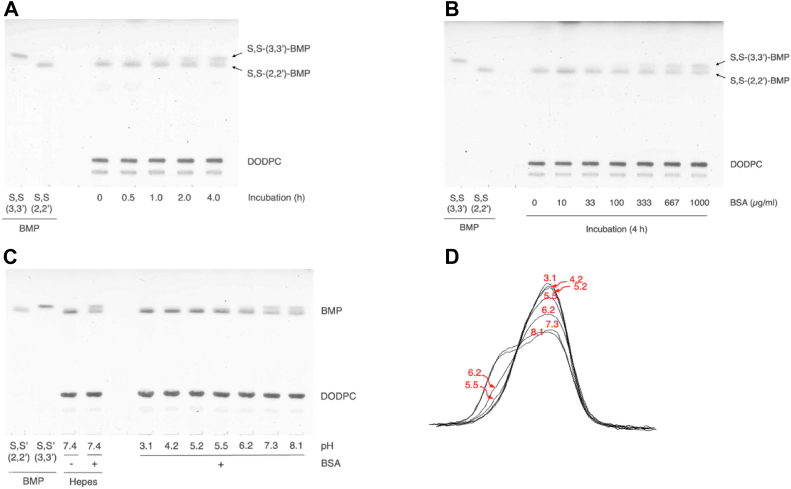
Time, BSA concentration, and pH dependence of isomerization of S,S-(2,2′-dioleoyl)-BMP in the presence of BSA. Representative TLC showing the time (A), BSA concentration (B), and pH dependence of the conversion of DODPC/S,S-(2,2′-diC_18:1_)-BMP to DODPC/S,S-(3,3′-diC_18:1_)-BMP by BSA. In panel, the reaction mixture containing DODPC/S,S-(2,2′-diC_18:1_)-BMP (molar ratio 7:3, 7.2 μM as phospholipid) was incubated at pH 7.4 in the presence of 0.667 mg/ml BSA for 0, 0.5, 1, 2 and 4 h at 37°C. The reaction products were detected as described in Fig. 1. In panel B, the reaction mixture containing DODPC/S,S-(2,2′-diC_18:1_)-BMP was incubated at pH 7.4 in the presence of different concentrations of BSA, 0, 33.3, 100, 333, 667, and 1,000 μg/ml for 4 h at 37°C. The reaction products were detected as described in Fig. 1. In panel C, DODPC/S,S-(2,2′-diC_18:1_)-BMP (molar ratio 2:1, 6.3 μM as phospholipid) liposomes were dispersed in citric acid-Na_2_HPO_4_ buffer at different pH in the presence of 667 μg/ml BSA and incubated for 4 h at 37°C. The reaction products were detected as described in Fig. 1. Individual lanes (pH 3.1–8.1) of the plate were scanned and the resultant curves were shown (panel D). BMP, bis(monoacylglycero)phosphate; DODPC, 1,2-di-*O*-(9Z-octadecenyl)-*sn*-glycero-3-phosphocholine.

To investigate further the isomerization of S,S-(2,2′-diC_18:1_)-BMP to S,S-(3,3′-diC_18:1_)-BMP, DODPC/S,S-(2,2′-diC_18:1_)-BMP liposomes were incubated at different times at 37°C in the presence of BSA at pH 7.4 (Fig. 2A). In addition, DODPC/S,S-(2,2′-diC_18:1_)-BMP liposomes were incubated for 4 h at 37°C with different concentrations of BSA at pH 7.4 (Fig. 2B). Under these conditions the isomerization of S,S-(2,2′-diC_18:1_)-BMP to S,S-(3,3′-diC_18:1_)-BMP was increased in a time-dependent and BSA concentration-dependent manner. This isomerization was observed at pH 5.5, gradually accelerated with increasing pH, and the rate of conversion of 2,2'-(S,S-diC_18:1_)-BMP to 3,3'-(S,S- diC_18:1_)-BMP reached a near maximum at neutral pH (Fig. 2C, D).

### Effect of the molar ratio of BMP in liposomes on the conversion of S,S-(2,2′-diC_18:1_)-BMP to S,S-(3,3′-diC_18:1_)-BMP in the presence of BSA

Intracellular BMPs are localized and abundant in late endosomes and lysosomes, accounting for ∼15% of the total phospholipids in late endosomes and endolysosomes in baby hamster kidney cells (3, 18). The molar fraction of BMP intraluminal vesicles (ILVs) in these acidic compartments is substantially higher and reaches ∼70%. We sought to verify whether the molar ratio of BMP in phospholipid membrane affects the conversion of S,S-(2,2′-diC_18:1_)-BMP to S,S-(3,3′-diC_18:1_)-BMP promoted by BSA.

**Fig. 3:**
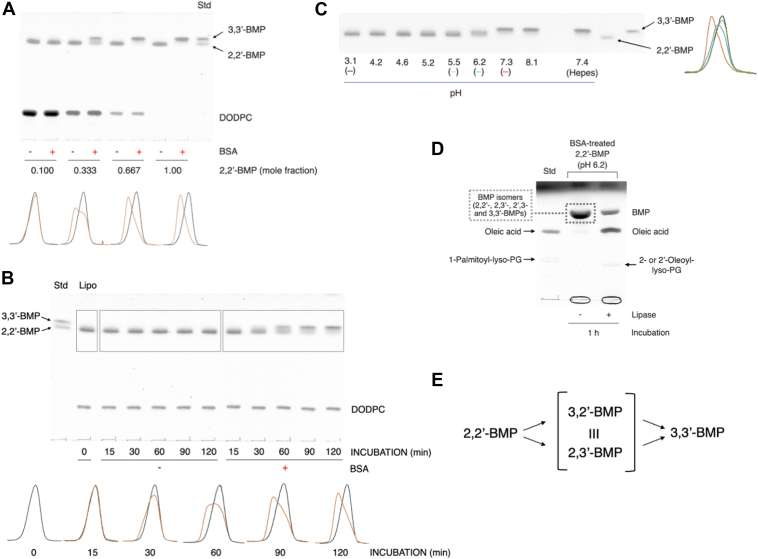
Effect of the molar ratio of BMP in liposomes on isomerization of S,S-(2,2′-diC_18:1_)-BMP in the presence of BSA. Representative TLC showing the BMP mol fraction dependence in liposomes (A) and the time (B) and pH (C) dependence in liposomes with high BMP content for the conversion of DODPC/S,S-(2,2′-diC_18:1_)-BMP to DODPC/S,S-(3,3′-diC_18:1_)-BMP by BSA. A: Liposomes consisting of DODPC and S,S-(2,2′-diC_18:1_)-BMP (molar ratio 9:1, 7:3, 3:7, and 0:1, 7.2 μM as phospholipid) were incubated with 0.667 mg/ml BSA in 50 mM sodium chloride/15.7 mM Hepes (pH 7.4) for 4 h at 37°C. In panel B, DODPC/S,S-(2,2′-diC_18:1_)-BMP liposomes (3:7 molar ratio, 7.2 μM as phospholipid) were incubated with 0.667 mg/ml BSA at 37°C for 0, 15, 30, 60, 90, and 120 min in 50 mM sodium chloride/15.7 mM Hepes (pH 7.4). The reaction products were detected as described in Fig. 1. Individual lanes of the plate were scanned, and the resultant curves were shown at the bottom. Re-evaluation of the pH dependence of the BSA-promoted isomerization of S,S-(2,2′-diC18:1)-BMP and identification of the BMP isomers generated by isomerization. In this study, 100% S,S-(2,2′-diC_18:1_)-BMP liposomes were used to re-examine the pH dependence of the conversion of 100% S,S-(2,2′-diC_18:1_)-BMP in the presence of BSA. As shown in panel C in Fig. 2, S,S-(2,2′-diC_18:1_)-BMP (2.1 μM as phospholipid) were dispersed in citric acid-Na_2_HPO_4_ buffer at different pH in the presence of 0.667 mg/ml BSA and incubated for 1 h at 37°C. Panel D is a representative TLC showing the production of lyso-PG from the isomerization reaction mixture treated with *sn*-1,3-specific lipase. The reaction products were detected as described in Fig. 1. Selected lanes (pH 3.1 (black), 5.5 (green), 6.2 (blue), and 7.3 (red)) of the plate were scanned and the resultant curves were shown (panel C, right inset). In panel D, the lipids extracted from the reaction products obtained from 1 h incubation in panel C (pH 6.2) were treated with or without *sn*-1,3-specific lipase from *Rhizomucor miehei* for 1 h at 37°C. Panel E shows a model for the isomerization of S,S-(2,2′-diC_18:1_)-BMP to S,S-(3,3′-diC_18:1_)-BMP. BMP, bis(monoacylglycero)phosphate; DODPC, 1,2-di-*O*-(9Z-octadecenyl)-*sn*-glycero-3-phosphocholine.

**Fig. 4:**
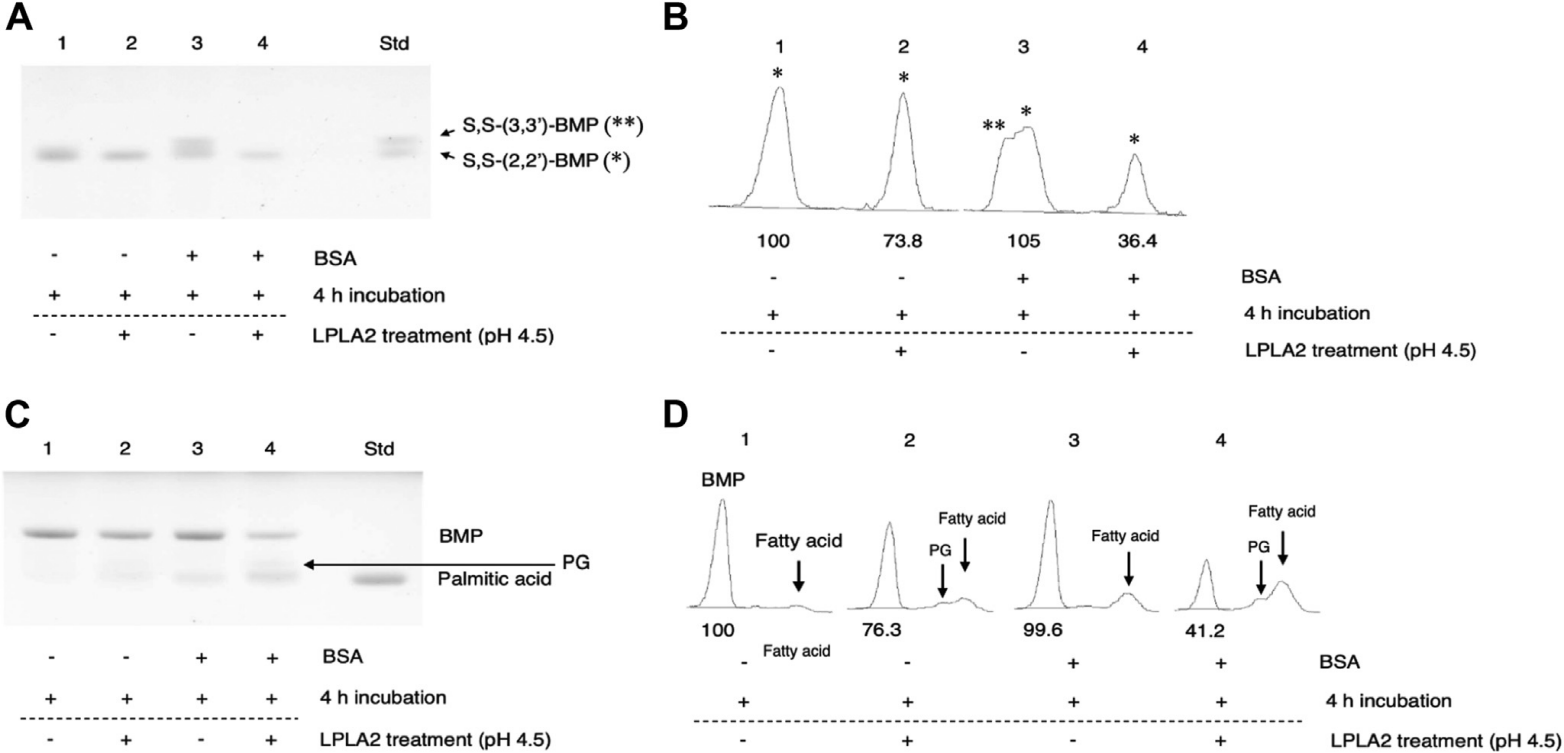
Isomerization and degradation of S,S-(2,2′-dioleoyl)-BMP by BSA/hLPLA2. Representative TLC showing isomerization and degradation of S,S-(2,2′-diC_18:1_)-BMP by BSA and hLPLA2, respectively. In panel A and B, the reaction mixtures containing DODPC/S,S-(2,2′-diC_18:1_)-BMP liposomes (molar ratio 7:3, 7.2 μM as phospholipid) was incubated at pH 7.4 in the absence or presence of 0.667 mg/ml BSA were incubated for 4 h at 37°C. After incubation, the pH of these reaction mixtures was adjusted to 4.5 and then the pH-adjusted reaction mixtures were treated with or without hLPLA2 for 1 h at 37°C. The resultant products were extracted and detected as described in Fig. 1. In panel A, the plate was developed in a solvent solution consisting of C/M/acetone/acetic acid/water (45:15:10:10:2, v/v). In panel C, the plate was developed in a solvent solution consisting of C/M/28% ammonium solution (65:35:8, v/v). BMP profiles of each lane taken in the J images of the left panels of A and C are placed on the right panels, B and D, respectively. Numbers below the profiles indicate the relative value of BMP areas. BMP, bis(monoacylglycero)phosphate; DODPC, 1,2-di-*O*-(9Z-octadecenyl)-*sn*-glycero-3-phosphocholine.

S,S-(2,2′-diC_18:1_)-BMP/DODPC liposomes with different molar ratios were prepared and incubated for 4 h under at pH 7.4 in the presence or absence of BSA (Fig. 3A). The conversion induced by BSA was dependent on the molar ratio of S,S-(2,2′-diC_18:1_)-BMP to DODPC in liposomes (Fig. 3A). Based on the peak shift of S,S-(2,2′-diC_18:1_)-BMP to S,S-(3,3′-diC_18:1_)-BMP over time, the conversion rate of liposomes with a molar ratio of 0.667 was estimated to be approximately three times higher than that of liposomes with a molar ratio of 0.333 (Fig. 3B). The composition of the latter liposomes is similar to that used in other experiments (Figs. 1, 2 and 4). In general, S,S-(2,2′-diC_18:1_)-BMP liposomes containing no or very small amounts of other lipids is a convenient tool for assaying the isomerization of S,S-(2,2′-diC_18:1_)-BMP.

### Re-evaluation of the pH dependence of the BSA-promoted isomerization of S,S-(2,2′-diC_18:1_)-BMP

We reevaluated the pH dependence of the BSA-promoted conversion of S,S-(2,2′-diC_18:1_)-BMP to S,S-(3,3′-diC_18:1_)-BMP using 100% S,S-(2,2′-diC_18:1_)-BMP liposomes that did not contain other lipids (Fig. 3C). The conversion was suppressed under acidic conditions between pH 3.1 and 5.2 (Fig. 3C, pH 3.1, right inset (black)), was observed at pH 5.5 (Fig. 3C, right inset (blue)), was prominent at pH 6.2 (Fig. 3C, right inset (green)), and was nearly complete at neutral (Fig. 3C, pH 7.3, right inset (red)) and weakly alkaline (Fig. 3C) conditions. The pH dependence was more evident than that observed in Figs. 2C, D.

In this assay, a broad band was observed between (2,2′-diC_18:1_)-BMP and S,S-(3,3′-diC_18:1_)-BMP bands when the liposomes were treated at pH 6.2 (Fig. 3C, (green)). The lipid extracts obtained from this reaction products were treated with *sn*-1,3-specific lipase and analyzed by TLC (Fig. 3D). Both oleic acid and 2-oleoyl-lyso-PG were produced at pH 6.2 by the lipase treatment (Fig. 3D). These results indicate that the reaction products at pH 6.2 must contain BMP isomers including (2,2′-diC_18:1_)-BMP, S,S-(2,3′-diC_18:1_)-BMP, S,S’-(3,2′-diC_18:1_)-BMP and S,S-(3,3′-diC_18:1_)-BMP (Fig. 3E) supporting the presence of S,S-(2,3′-diC_18:1_)-BMP, S,S’-(3,2′-diC_18:1_)-BMP. There is no stereochemical difference between S,S-(2,3′-diC_18:1_)-BMP and S,S’-(3,2′-diC18:1)-BMP, which are identical BMP molecules.

### Degradation of the S,S-(3,3′-diC_18:1_)-BMP converted from S,S-(2,2′-diC_18:1_)-BMP by hLPLA2

LPLA2 hydrolyses BMP isomers with distinct substrate specificity (17). We observed that the deacylation rate of S,S-(3,3′-diC_18:1_)-BMP by LPLA2 was much higher than that of S,S-(2,2′-diC_18:1_)-BMP, which is a poor substrate for LPLA2. Therefore, the conversion of S,S-(2,2′-diC_18:1_)-BMP to S,S-(3,3′-diC_18:1_)-BMP should facilitate the overall degradation of S,S-(2,2′-diC_18:1_)-BMP by LPLA2. In this study, we confirmed that the S,S-(3,3′-diC_18:1_)-BMP converted from S,S-(2,2′-diC_18:1_)-BMP in the presence of BSA is preferentially degraded as a substrate for LPLA2.

S,S-(2,2′-diC_18:1_)-BMP in liposomes, pre-incubated for 4 h without BSA at pH 7.4 and acidified with sodium acetate (pH 4.5), remained stable after an additional 1 h incubation (Fig. 4A, B, lane 1), but was slightly reduced by incubation with hLPLA2 (Fig. 4A, B, lane 2). In addition, a reaction mixture containing S,S-(3,3′-diC_18:1_)-BMP, S,S-(2,3′-diC_18:1_)-BMP and S,S-(2,2′-diC_18:1_)-BMP prepared by incubating for 4 h with BSA at pH 7.4 was acidified with sodium acetate and treated with hLPLA2 (Fig. 4A, B, lane 4) or without hLPLA2 (Fig. 4A, B, lane 3). The S,S-(3,3′-diC_18:1_)-BMP and S,S-(2,3′-diC_18:1_)-BMP formed by BSA treatment was completely degraded with hLPLA2 (Fig. 4A, B, lane 4), whereas the S,S-(2,2′-diC_18:1_)-BMP in the reaction mixture was poorly degraded with hLPLA2 (Fig. 4A, B, lane 4). At the same time, fatty acid release associated with LPLA2 reaction was detected in the samples treated with hLPLA2. The release of fatty acid by LPLA2 from BSA-treated liposomes was clearly greater than that from non-BSA–treated liposomes even though the BSA-treated liposomes contained an unknown fatty acid like band as background, which might have originated from BSA (Fig. 4C, D, lane 3 and 4). In addition, slight but detectable formation of PG by LPLA2 was observed in the reaction products (Fig. 4C, D, lane 2 and lane 4).

**Supplemental fig 1:**
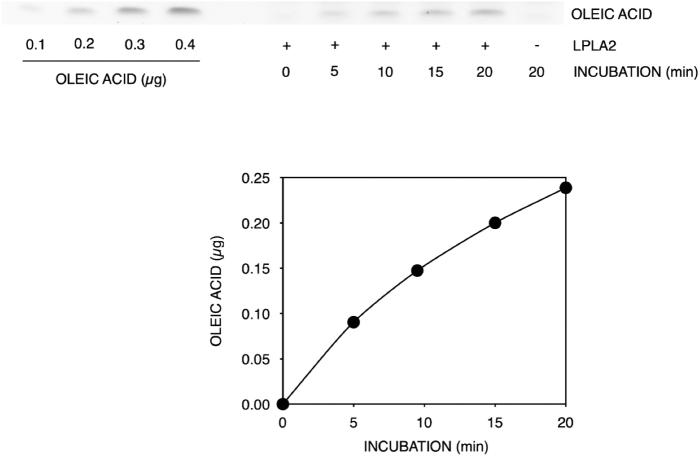


The initial degradation rate of S,S-(3,3′-diacyl)-BMP by hLPLA2 at pH 7.4 (1.3 μmol/min/mg protein) (Supplemental Fig. S1) was approximately 10-fold lower than that at pH 4.5 (17), but the degradation rate of the former was comparable to that of DOPC at pH 4.5 (23).

### Isomerization of S,S-(2,2′-diC_18:1_)-BMP by proteins other than BSA

**Fig. 5:**
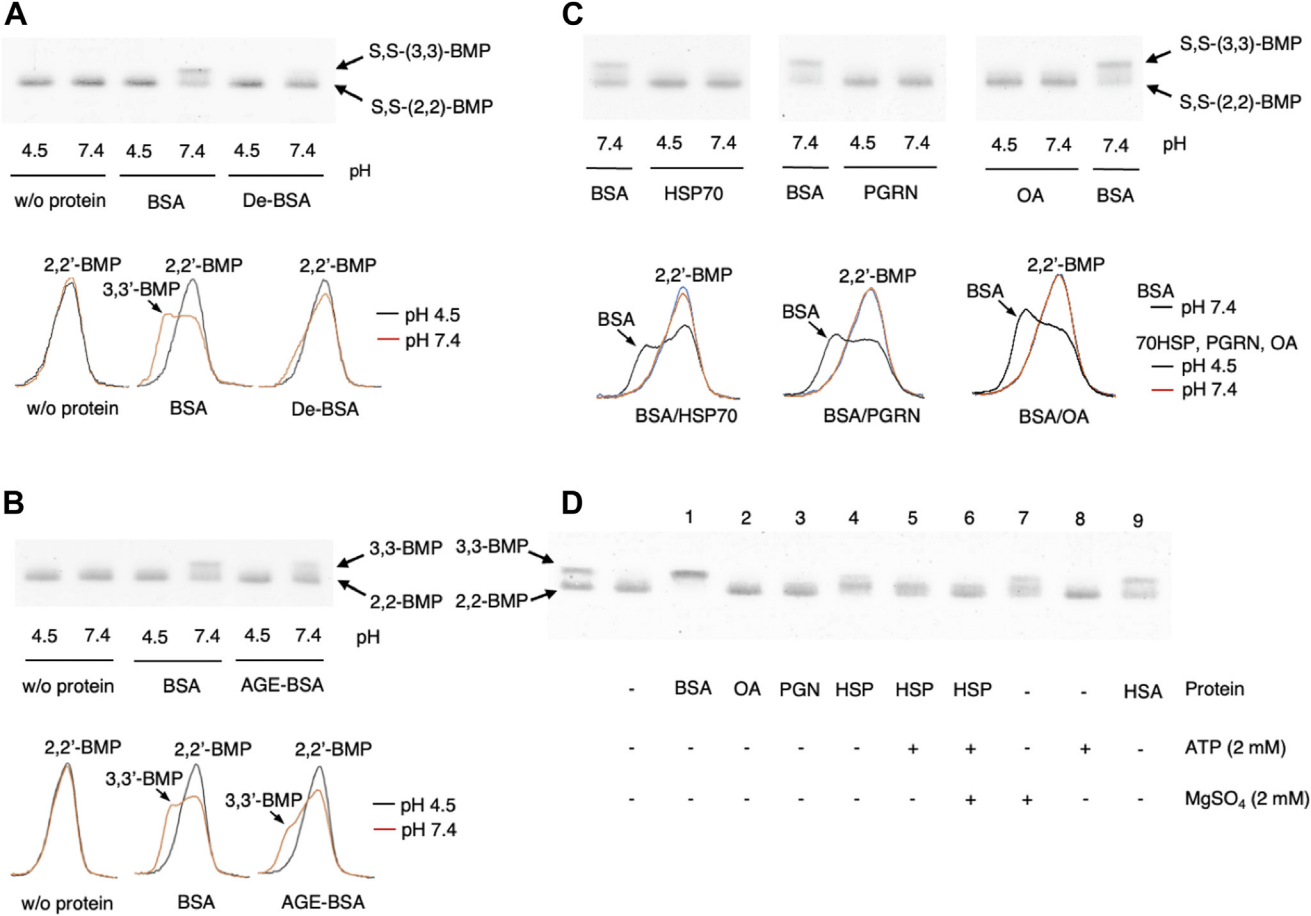
Isomerization of S,S-(2,2′-dioleoyl)-BMP by other proteins. Representative TLC showing S,S-(2,2′-diC_18:1_l)-BMP isomerization by heat-denatured BSA (A), AGE-BSA (B), HSP70, progranulin (PGRN) and ovalbumin (OA) (C). The isomerization of S,S-(2,2′-diC_18:1_)-BMP was investigated using proteins other than intact BSA. The reaction mixtures contained liposomes (6.3 μM as phospholipid) consisting of DODPC/S,S-(2,2′-diC_18:1_)-BMP (molar ratio 2:1), 50 mM sodium chloride, 15.7 mM sodium acetate (pH 4.5), or 15.7 mM Hepes (pH 7.4) and were incubated for 4 h at 37°C in the presence or absence of 0.667 mg/ml of BSA or heat-modified BSA (De-BSA) (panel A), and AGE-BSA (panel B). In panel C, the reaction mixtures containing liposomes (DODPC/BMP (7:3), 7.2 μM as phospholipid) were incubated with 0.667 mg/ml of human recombinant HSP70, human recombinant PGRN or OA under acidic or neutral conditions as described above. The de-BSA was prepared by heating BSA in 100 mM sodium acetate (pH 8.6) at 65°C for 1 h. The reaction products shown on each plate were scanned and the resulting curves were overlaid with black, blue, or red lines. In panels A and B, the lines obtained from pH 4.5 and pH 7.4 are shown in black and red, respectively. In panel C, the line obtained from BSA (pH 7.4) is shown in black. For other proteins, the lines obtained from pH 4.5 and 7.4 are shown in blue and red, respectively. Representative TLC showing S,S-(2,2′-diC_18:1_)-BMP isomerization by BSA, OA, PGRN, HSP70, and human serum albumin using 100% BMP liposomes (D). In panel D, 100% of S,S-(2,2′-diC_18:1_)-BMP liposomes were incubated with 0.3 mg/ml BSA, OA, PGRN, HPS (HSP70), human serum albumin, or 2 mM MgSO_4_ for 6 h at 37°C. In addition, HSP70 was incubated in the presence of 2 mM ATP (lane 5) or 2 mM ATP/2 mM MgSO_4_ (lane 6). AGE, advanced glycation end product; BMP, bis(monoacylglycero)phosphate; DODPC, 1,2-di-*O*-(9Z-octadecenyl)-*sn*-glycero-3-phosphocholine.

BSA is well characterized as a carrier of various small molecules, including sterols, bilirubin, fatty acids, lyso-phospholipids, thyroid hormones, nucleotides, and minerals. The tertiary structure of BSA is important for BSA to act in this function. BSA treated at 65^o^C and pH 8.6 for 1 h was used as heat-denatured BSA (de-BSA). The isomerization of S,S-(2,2′-diC_18:1_)-BMP to S,S-(3,3′-diC_18:1_)-BMP was enhanced at pH 7.4 in the presence of intact BSA. In contrast, BSA-induced isomerization was markedly suppressed by heat treatment of BSA (Fig. 5A, de-BSA).

**Supplemental fig 2:**
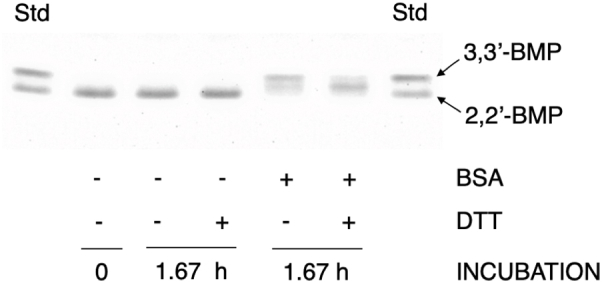


We also investigated chemically modified BSAs. Advanced glycation end product-BSA and DTT-treated BSA, which contain modified secondary structures induced by the glycation at lysine residues (24) and the S-S bridge exchange reaction (25), respectively, partially reduced their ability to promote S,S-(2,2′-diC_18:1_)-BMP isomerization (Fig. 5B, Supplemental Fig. S2). These results indicate that the intact structure of BSA is required to for full isomerization of S,S-(2,2′-diC_18:1_)-BMP.

In addition, other proteins that interact with phospholipid membranes, such as HSP70 (26), progranulin (27), and ovalbumin (28), also failed to reproduce the isomerization of S,S-(2,2′-diC_18:1_)-BMP enhanced by BSA (Fig. 5C, third, second, and first panels from the right).

These studies were carried out using liposomes with a BMP content of 33 mol%. As shown above, the isomerization of S,S-(2,2′-diC_18:1_)-BMP by BSA was enhanced with an increase in the S,S-(2,2′-diC_18:1_)-BMP fraction in the liposomes (Fig. 3A). In other words, the higher the ratio of S,S-(2,2′-diC_18:1_)-BMP/DODPC, the greater the isomerization of S,S-(2,2′-diC_18:1_)-BMP. To fully assess whether the proteins other than BSA also promote the isomerization, 100% S,S-(2,2′-diC_18:1_)-BMP liposomes were used in the following studies.

Under these conditions but unlike ovalbumin or progranulin, HSP70 showed a moderate increase in isomerization under neutral conditions (Fig. 5D, lane 4). Also, HSA (Fig. 5D, lane 9) displayed less isomerization than BSA (Fig. 5D, lane 1). According to the vendor’s specification sheet, the BSA used in this study was fatty acid free protein prepared from fraction V. In contrast, we detected fatty acid in the HAS used in this study (data not shown). In addition, the BSA pretreated with oleic acid reduced the promotion rate of the isomerization (data not shown). Thus, binding of fatty acids to serum albumin might possibly affect its ability to isomerize S,S-(2,2′-diC_18:1_)-BMP.

HSP70 is a protein having a nucleotide-binding domain (NBD) that plays an important role in interacting with lipid membranes (29). The isomerization by HSP70 was reduced in the presence of ATP (Fig. 5D, lane 5). This inhibitory effect by ATP was enhanced in the presence of Mg^2+^ (Fig. 5D, lane 6), although Mg^2+^ by itself promoted the isomerization of S,S-(2,2′-diC_18:1_)-BMP (Fig. 5D, lane 7). Lysosomes are acidic compartments that contain BMP molecules and are involved in heavy metal metabolism and homeostasis (30, 31). Heavy metals such as iron are involved in oxidative stress and redox reactions within cells and are cytotoxic when accumulated. We therefore decided to examine whether metal ions other than Mg^2+^ promote the isomerization of S,S-(2,2′-diC_18:1_)-BMP.

### Isomerization of S,S-(2,2′-diC_18:1_)-BMP by metal ions

**Fig. 6:**
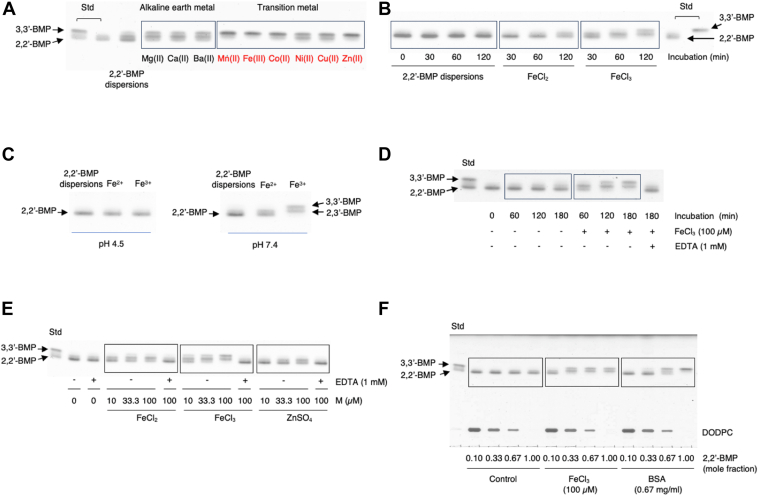
Isomerization of S,S-(2,2′-dioleoyl)-BMP by metal ions. Representative TLCs showing the isomerization of S,S-(2,2′-diC_18:0_)-BMP by metal ions (A–E). In studies in panels A–E, 100% S,S-(2,2′-diC_18:0_)-BMP (10.9 μM as phospholipid) liposomes were used. In panel A, S,S-(2,2′-diC_18:0_)-BMP liposomes were incubated with 2 mM of various metal compounds for 13 h at 37°C. For Mg (II), Ca (II), Ba (II), Mn (II), Fe (III), Co (II), Ni (II), Cu (II), and Zn (II) indicate MgSO_4_, CaCl_2_, BaCl_2_, MnCl_2_, FeCl_3_, CoCl_2_, NiSO_4_, CuSO_4_, and ZnCl_2_, respectively. In panel B, S,S-(2,2′-diC_18:0_)-BMP liposomes were incubated with 2 mM of FeCl_2_ or FeCl_3_ for 30, 60, or 120 min. In panel C, S,S-(2,2′-diC_18:0_)-BMP liposomes were incubated with 2 mM of FeCl_2_ or FeCl_3_ at pH 4.5 (20 mM sodium acetate buffer) for 6 h or at pH 7.4 (20 mM Hepes buffer) for 2 h. In panel D, S,S-(2,2′-diC_18:0_)-BMP liposomes were incubated with 100 μM FeCl_3_ for 60, 120, or 180 min. For the 180-min incubation, the reaction mixture was incubated in the presence or absence of 1 mM EDTA. In panel E, S,S-(2,2′-diC_18:0_)-BMP liposomes were incubated with 10, 33.3, or 100 μM FeCl_2_, FeCl_3_, or ZnSO4 for 3 h at 37°C. For incubations with 100 μM of each metal compound, each reaction mixture was incubated in the presence or absence of 1 mM EDTA. In panel F, liposomes consisting of DODPC and S,S-(2,2′-diC_18:1_)-BMP (molar ratio 9:1, 7:3, 3:7, or 0:1, 10.9 μM S,S-(2,2′-diC_18:1_)-BMP as phospholipid) were incubated with 100 μM FeCl_3_ or 0.67 mg/ml BSA for 3 h at 37°C. All plates were developed in a solvent system consisting of C/M/acetone/acetic acid/water, 45:15:10:10:2, v/v). BMP, bis(monoacylglycero)phosphate; C/M, chloroform/methanol; DODPC, 1,2-di-*O*-(9Z-octadecenyl)-*sn*-glycero-3-phosphocholine.

Liposomes containing 100% S,S-(2,2′-diC_18:1_)-BMP liposomes were used to study the effects of metal ions on the isomerization of S,S-(2,2′-diC_18:1_)-BMP. Two mM of each metal ion was incubated with S,S-(2,2′-diC_18:1_)-BMP liposomes under neutral conditions overnight. The transition metal ions, Mn^2+^, Fe^3+^, Co^2+^, Ni^2+^, Cu^2+^, and Zn^2+^, were stronger promotors of S,S-(2,2′-diC_18:1_)-BMP isomerization than the alkaline earth metal ions, Mg^2+^, Ca^2+^, and Ba^2+^ (Fig. 6A). Incubation of S,S-(2,2′-diC_18:1_)-BMP overnight in the presence of Mn^2+^, Fe^3+^, Co^2+^, and Zn^2+^ markedly enhanced the conversion of S,S-(2,2′-diC_18:1_)-BMP to S,S-(3,3′-diC_18:1_)-BMP (Fig. 6A).

Iron is a major transition metal normally found in lysosomes. Abnormal iron metabolism and iron-redox in lysosomes is strongly associated with ferroptosis (32). The effect of Fe^2+^ on the isomerization of S,S-(2,2′-diC_18:1_)-BMP was compared with that of Fe^3+^.

S,S-(2,2′-diC_18:1_)-BMP was time dependently converted to other isomers in the presence of 1 mM FeCl_2_ or FeCl_3_ (Fig. 6B). Isomerization of S,S-(2,2′-diC_18:1_)-BMP in the presence of Fe^3+^ to S,S-(2,3′- or 3,2′-diC_18:1_)-BMP and S,S-(3,3′-diC_18:1_)-BMP was observed at 120 min after initiation of the reaction. By contrast, in the presence of Fe^2+^, more than half of S,S-(2,2′-diC_18:1_)-BMP remained as the major isomer in the reaction mixture even at 120 min (Fig. 6B). The isomerization by both ions was markedly suppressed under acidic conditions as was observed with BSA (Fig. 6C).

We observed the generation of some insoluble materials if the reaction contained mM concentrations of iron ions under neutral conditions, possibly iron hydroxides. To minimize the formation of these insoluble substances, iron ion concentrations below 100 μM were used in subsequent studies. At these lower concentrations conversion of S,S-(2,2′-diC_18:1_)-BMP to other BMP isomers proceeded in a time-dependent manner in the presence of 100 μM FeCl_3_ at pH 7.4 (Fig. 6D). Studies comparing the effects of zinc and iron ions were performed with incubations as short as 3 h. In all cases, the conversion of S,S-(2,2′-diC_18:1_)-BMP to other BMP isomers occurred in a concentration-dependent manner (Fig. 6E). The rank order of isomerization efficiency was Fe^3+^> Fe^2+^> Zn^2+^. Fe^3+^ was much more efficient than other Fe^2+^ and Zn^2+^. In addition, the isomerization by these ions was completely inhibited in the presence of EDTA (Fig. 6D, E). The efficiency of isomerization by Fe^3+^ for 3 h varied depending on the molar ratio of BMP/DODPC in the liposomes (Fig. 6F). Unlike BSA, enhanced isomerization occurred at a lower molar ratio of 0.10 for S,S-(2,2′-diC_18:1_)-BMP/DODPC, reaching a near plateau at 0.33 (Fig. 6F).

### Acyl migration of S,S’-(2,2′-diacyl)-BMP within cells

To investigate whether S,S’-(2,3′- and 3,3′-diacyl)-BMPs exist endogenously and whether their levels in cells can be modified, we used BMP-rich RAW-264.7 cells and *sn*-1,3-specific lipase. RAW-264.7 cells were treated for 24 and 48 h with or without CHL or ammonium chloride (NH_4_Cl), lysosomotropic amines and generally used to increase the pH of acidic compartments.

**Fig. 7:**
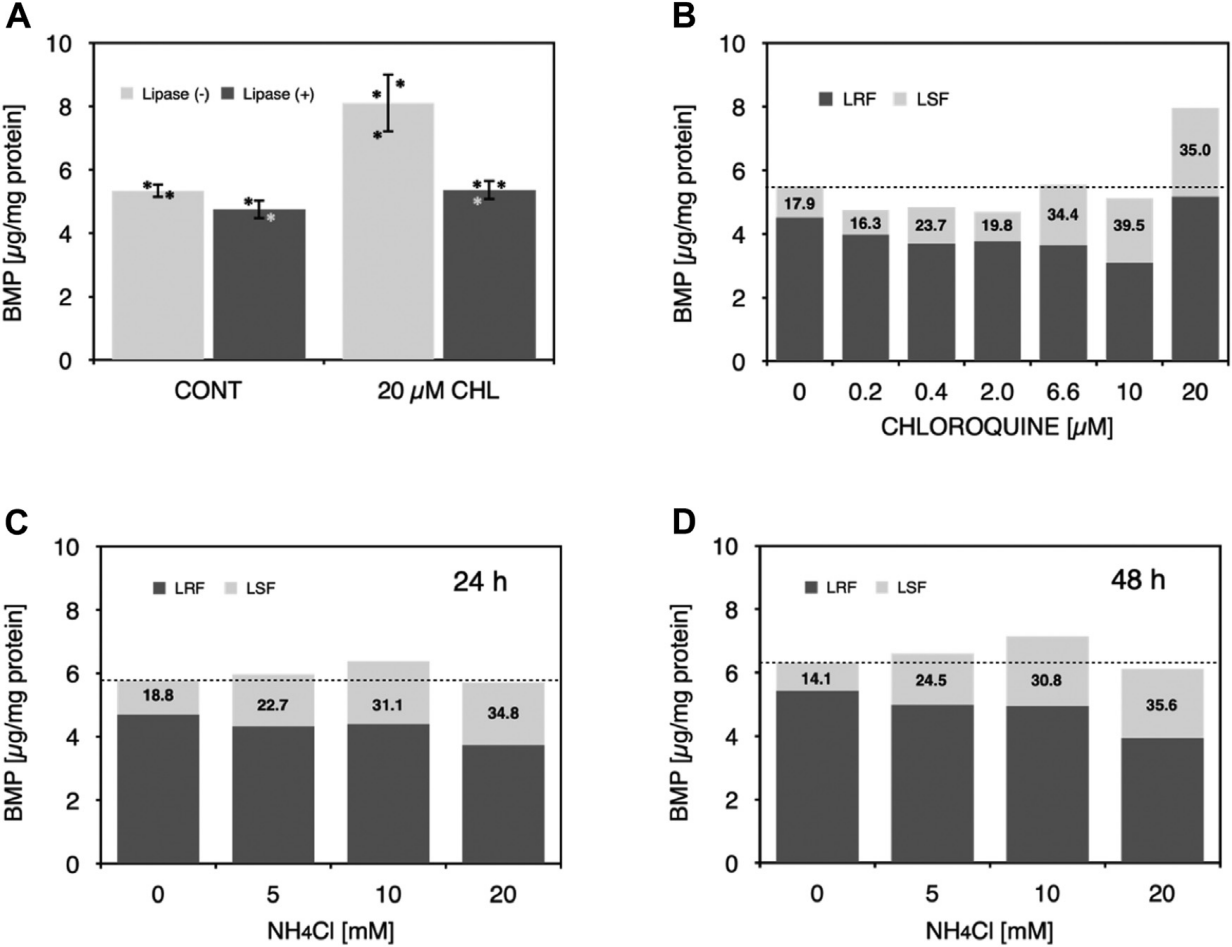
Lipase-sensitive BMPs in RAW 264.7 cells. The lipids extracted from the cell homogenates (90 μg of protein) obtained from Raw 264.7 cells were dispersed into 500 μl of 135 mM NaCl/20 mM Hepes (pH 7.4) by a probe-type sonicator for 8 min while cooing using an ice water bath. The resultant dispersions were treated with or without *sn*-1,3-specific lipase from *Rhizomucor miehei* for 1 h at 37°C. After treatment, the lipids were extracted and detected as shown in Fig. 1. The plate was developed in a solvent solution consisting of C/M/28% ammonium solution (65:35:5, v/v) and scanned as described in the Materials and methods. In panel A, the cells were treated with or without 20 μM CHL for 24 h at 37°C. The panel shows BMP levels of Raw 264.7 cell homogenates with or without treatment by *R. miehei* lipase. Values represent the mean + SD. (n = 2 for control, n = 3 for CHL treatment). The data are representative of two experiments with similar results. In panel B, the cells were treated with different concentrations of CHL, 0, 0.2, 0.4, 2.0, 6.6, 10, and 20 μM) for 48 h. In panel C and D, the cells were treated with different concentrations of NH_4_Cl, 0, 5, 10, and 20 mM) for 24 and 48 h, respectively. LSF and LRF indicate lipase-sensitive fraction and lipase-resistant fraction, respectively. For Fig. 7B–D, two similar experiments were performed separately, the results from a representative individual experiment is shown in Fig. 7B–D. BMP, bis(monoacylglycero)phosphate; C/M, chloroform/methanol; NH_4_Cl, ammonium chloride.

**Supplemental fig 3:**
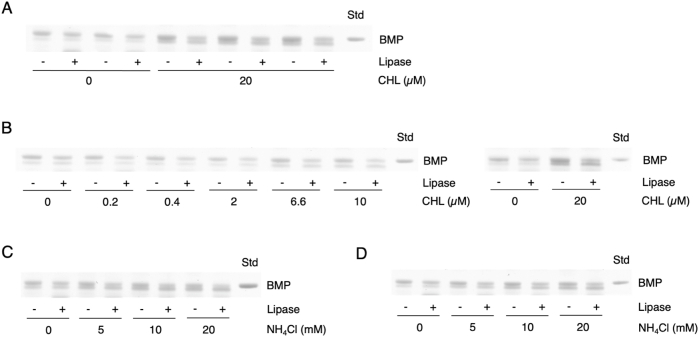


CHL is a known cationic amphiphilic compound that is trapped in acidic compartments, increases in luminal pH and induces phospholipidosis (33). First, we confirmed that CHL did not induce cell toxicity to our cultured cells in the concentration range from 0 to 20 μM. The increase of pH in the acidic compartment by CHL treatment occurs rapidly (34, 35). RAW-264.7 cells were treated with or without 20 μM CHL for 24 h. As shown in Fig. 7A and Supplemental Fig. S3A, total BMP content in CHL-treated cell homogenates was higher than in CHL-untreated cell homogenates. Also, in both groups, total BMP content was significantly reduced by treatment with *sn*-1,3-specific lipase (Fig. 7A and Supplemental Fig. S3A). As a result, 11.1 ± 1.99% of the total BMP in CHL-untreated cells was *sn*-1,3-specific lipase-sensitive fraction (LSF), whereas 36.4 ± 4.59% was LSF in CHL-treated cells. (Fig. 7A).

RAW-264.7 cells were also treated with different concentrations of CHL (0, 0.2, 0.4, 6.6, 10, and 20 μM) for 48 h. At lower concentrations, total BMP levels in CHL-treated cells were slightly lower than that in control cells up to 10 μM CHL (Fig. 7B, Supplemental Fig. S3B). However, the total BMP level in the cells treated with 20 μM appeared to be significantly higher than in control cells (Fig. 7B, Supplemental Fig. S3B). In addition, a substantial increase of the LSF of BMPs in the CHL-treated cells was observed at 6.6, 10, and 20 μM CHL administrations (Fig. 7B, Supplemental Fig. S3B). These results suggest that the increase in the LSF of BMPs in CHL-treated cells does not necessarily correlate with CHL-induced phospholipidosis but is associated with an increase in pH in acidic compartments.

### NH_4_Cl–treated RAW-264.7 cells

To confirm that the magnitude of LSF is independent of phospholipidosis, RAW-264.7 cells were treated with different concentrations of NH_4_Cl, which does not induce phospholipidosis, at (0, 5, 10, and 20 mM) for 24 and 48 h (Fig. 7C, Supplementary Fig. S3C, D). The cells appeared healthy during treatment. Total BMP content measured in cell homogenates did not differ between control and NH_4_Cl-treated cells. The control cells consistently contained approximately 15% LSF (Fig. 7). This LSF was increased by NH_4_Cl in a dose-dependent manner up to 35% (Fig. 7C, D) and reached equilibrium with the *sn*-1,3-specific lipase-resistant fraction 24 h after NH_4_Cl administration. These results confirm that the increase of LSF of BMPs associated with increased pH in acidic compartments is independent of cationic amphiphilic drug–induced phospholipidosis.

## Discussion

We report in this paper the important finding that there is regulated conversion of S,S-(2,2′-diacyl)-BMP to S,S-(3,3′-diacyl)-BMP. BMPs have been a subject of interest due to their unique subcellular distribution and atypical structure. Functional studies on BMPs have suggested important roles in lysosomal structure and regulation of catabolic hydrolases. Because S,S-(2,2′-diacyl)-BMP has been regarded as the sole biologically active and naturally occurring BMP, most of these studies have not determined if there are distinct roles for the S,S-(2,2′-diacyl)-BMP and S,S-(3,3′-diacyl)-BMP isomers. A notable exception is the work by Matsuo and colleagues who reported that Alix-dependent multivesicular liposomal membranes are formed in the presence of S,S-(2,2′-diacyl)-BMP and not S,S-(3,3′-diacyl)-BMP (36). The binding of other proteins, including acid sphingomyelinase and HSP70, to distinct BMP isomers does not appear to have been reported. S,S-(2,2′-diacyl)-BMP has a structure that is more tightly packed although less thermodynamically stable than S,S-(3,3′-diacyl)-BMP (37, 38). A recent publication reports that a sensing mechanism that targets the lysosome for autophagy by SPG20-ITCH is lysosomal membrane packing (39).

S,S-(2,2′-diacyl)-BMP structurally resembles 1-lyso-2-acyl-glycerophospholipids. 1-Lyso-2-acyl-phosphatidylcholine is spontaneously converted to the thermodynamically stable 1-acyl-2-lyso-acyl-phosphatidylcholine in a neutral aqueous solution (20, 21, 22, 40). The acyl migration from the C2-position to the C1-position likely occurs via a five-member ring intermediate formed by the nucleophilic attack of a lone pair of electrons of the oxygen of free hydroxyl group at the C1-position on the carbon of carbonyl group at the C2-position, the formation of which may be by exposure to an aqueous environment (40). The acyl migration rate is further enhanced in the presence of albumin under neutral conditions (20, 21, 22). Based on this structural similarity, we postulated that S,S-(2,2′-diacyl)-BMP is nonenzymatically isomerized to S,S-(3,3′-diacyl)-BMP, a BMP isomer that is thermodynamically more stable than S,S-(2,2′-diacyl)-BMP and a better substrate for LPLA2 (17).

In support of this interpretation, the conversion of S,S-(2,2′-diC_18:1_)-BMP to S,S-(3,3′-diC_18:1_)-BMP occurred under neutral conditions in the presence of BSA and was confirmed by deacylation with the *sn*-1,3-specific lipase fungal lipase (Fig. 1A–D, lane 4 upper band, and 1F, lane 6). Specifically, the diffuse staining component observed between S,S-(2,2′-diC_18:1_) and S,S-(3,3′-diC_18:1_) bands 4 h after treatment with BSA at pH 7.4 was sensitive to *sn*-1,3-specific lipase (Fig. 1F, lanes 5 and 6).

The isomerization promoted by BSA increased in time- and BSA concentration-dependent manners (Fig. 2A, B). Furthermore, the same isomerization was also pH-dependent (Fig. 2C), being observed at pH 5.5 and occurring at a maximum rate above pH 7 (Fig. 2C). These results indicate that BSA can efficiently promote the isomerization of S,S-(2,2′-diC_18:1_)-BMP to S,S-(3,3′-diC_18:1_)-BMP under neutral conditions.

As reported by Yokouchi *et al.*, the absorption of BSA into acidic lipid membranes induces a phase separation in liposomes, with subsequent formation of a temporary gap in the liposomal bilayer and increased permeability of the liposomal membrane (41). This BSA effect may increase exposure of S,S-(2,2′-diC_18:1_)-BMP molecule to an aqueous environment and contribute to promote the acyl migration of S,S-(2,2′-diC_18:1_)-BMP. Thus, the different mechanisms by which lyso-PC and BMP are exposed to the aqueous environment may be reflected as differences in the conversion half-lives of each lipid.

In late endosomes, the concentration of BMPs is approximately 15 mol% of the total lipid content of the organelle and is enriched up to 70 mol% of that of intraendosomal vesicles (3, 18). Interestingly, the conversion of S,S-(2,2′-diC_18:1_)-BMP to S,S-(3,3′-diC_18:1_)-BMP in the presence of BSA was enhanced by increasing the molar ratio of S,S-(2,2′-diC_18:1_)-BMP/DODPC in the liposomes (Fig. 3A). Dioleoyl-BMP vesicles are formed with a smaller diameter than either 1-palmitoyl-2-oleoyl-PG or 1-palmitoyl-2-oleoyl-PC when passed through extrusion membranes of equal size (42). Under acidic conditions, the phosphate group of BMP is a partially protonated and contributes to form more stable small vesicles. However, under neutral conditions, the phosphate of the same liposomes is deprotonated and generates repulsion between BMP molecules (43). Based on these reports, increasing the proportion of S,S-(2,2′-diC_18:1_)-BMP in liposomes should reduce the size of the vesicles, increases the curvature of the membrane, and destabilize the membrane under neutral conditions. This would facilitate the hydrophobic interaction between BSA and the liposome membrane, promoting the binding of BSA to the liposomes and increasing membrane permeability. These changes would accelerate the contact of BMP molecules with the aqueous environment, thereby facilitating the migration of the acyl group of S,S-(2,2′-diC_18:1_)-BMP. This was in fact observed as increasing the molar ratio of S,S-(2,2′-diC_18:1_)-BMP in S,S-(2,2′-diC_18:1_)-BMP/DODPC liposomes enhanced BSA-induced S,S-(2,2′-diC_18:1_)-BMP isomerization (Fig. 3A, B).

The pH dependence of the acyl migration of S,S-(2,2′-diC_18:1_)-BMP promoted by BSA measured using 30% S,S-(2,2′-diC_18:1_)-BMP liposomes persisted even when 100% S,S-(2,2′-diC_18:1_)-BMP liposomes were used (Figs. 2C and 3C). The reaction product obtained from 100% mol S,S-(2,2′-diC_18:1_)-BMP treated with BSA at pH 6.2 when treated with *sn*-1,3-specific lipase formed not only oleic acid but also 1-lyso-2-oleoyl-PG (Fig. 3D). Under the current reaction conditions, 1,3-specific lipase deacylates the acyl groups attached to *sn*-1 or 1′ and *sn*-3 or 3′ of dioleoyl-BMP. As shown in Fig. 1E, F, S,S-(3,3′-diC_18_)-BMP is completely deacylated by 1,3-specific lipase, but S,S-(2,2′-diC_18_)-BMP is not. Based on the substrate specificity of 1,3-specific lipase, the lyso-PG generated by 1,3-specific lipase from the BMP mixture formed from S,S-(2,2′-diC_18_)-BMP in the presence of BSA should be 1-lyso-2-acyl-PG or 1′-lyso-2′-acyl-PG, not 1-acyl-2-lyso-PG or 1′-acyl-2′-lyso-PG. In light of these observations and the transition profile of S,S-(2,2′-diC_18:1_)-BMP to S,S-(3,3′-diC_18:1_)-BMP observed in the presence of BSA (Fig. 3B), the isomerization of S,S-(2,2′-diC_18:1_)-BMP to S,S-(3,3′-diC18:1)-BMP likely proceeds via the S,S-2,3′-diC_18:1_ or S,S-3,2′-diC_18:1_)-BMP isomer as an intermediate (Fig. 3E). Stated differently, the acyl migration of the *sn*-2- and 2′-oleoyl groups to the *sn*-3 and 3′ positions does not occur simultaneously but rather in a stepwise manner (Fig. 1).

As predicted from the substrate specificity of LPLA2 for BMP isomers (17), when the S,S-(3,3′-diC_18:1_)-BMP formed from S,S-(2,2′-diC_18:1_)-BMP under neutral conditions in the presence of BSA was treated with hLPLA2 under acidic conditions, S,S-(3,3′-diC_18:1_)-BMP was preferentially deacylated over S,S-(2,2′-diC_18:1_)-BMP (Fig. 4A, B, lane 4). At the same time, the production of small but detectable amounts of PG was observed (Fig. 4C, D, lane 4). LPLA2 possesses dual enzyme activities, phospholipase A and transacylase (44, 45). In general, primary hydroxyl groups are better acyl acceptors than secondary hydroxyl groups (46, 47). Thus, one of the reaction products, 2-oleoyl-lysoPG, could react with the oleoyl-hLPLA2 intermediate as an acyl acceptor to produce dioleoyl-PG with S,S configuration. In fact, LPLA2 is observed to transfer the acyl group of diacyl-glycerophospholipids, including PG, to the *sn*-3′ position of PG and to the *sn*-2 or *sn*-3′ position of 1-acyl-lyso-PG in vitro (unpublished data). The former product is hemi BMP (R,S) and the latter product is PG (R,S) or BMP (R,S).

It is noteworthy that the deacylation rate of S,S-(3,3′-diacyl)-BMP by hLPLA2 at pH 7.4 was comparable to that of DOPC at pH 4.5 (Supplemental Fig. S1) (23), supporting the view that the reactivity of S,S-(3,3′-diacyl)-BMP as a substrate for LPLA2 under neutral conditions is biologically significant.

When S,S-(2,2′-diC_18:1_)-BMP isomerization was assayed with 100% BMP liposomes, HSP70 and HSA, unlike ovalbumin and progranulin, had a moderate ability to isomerize 2,2′-BMP compared to BSA (Fig. 5D). The lower observed S,S-(2,2′-diC_18:1_)-BMP isomerization by HSA could be due to fatty acid binding of HSA, which leads to changes in the protein's conformation and membrane affinity. HSP70 stabilizes lysosomes by binding to the endolysosomal anionic phospholipid BMP under acidic conditions, is an essential cofactor for lysosomal sphingomyelin metabolism (48). In addition, HSP70 has a NBD and its effect on isomerization was reduced in the presence of ATP or ATP/Mg^2+^ at neutral pH (Fig. 5D, lanes 5 and 6). The binding of HSP70 to lipid membranes containing BMP is reported to be reduced by ATP and ADP (29). The conformational change of HSP70 induced by the binding ATP at the NBD may affect the interaction between the protein and the liposomes (49). Taken together, to better promote protein-induced S,S-(2,2′-diC_18:1_)-BMP isomerization, not only the protein's ability to bind to lipid membranes but also the proper stereochemical structure and membrane-binding mode of the protein is required.

The unexpected finding that Mg^2+^ enhances the isomerization of S,S-(2,2′-diC_18:1_)-BMP to other BMP isomers (Fig. 5D, lane 7) prompted the investigation of other metal ions. The transition metal ions such as Mn^2+^, Fe^3+^, Co^2+^, Ni^2+^, Cu^2+^, and Zn^2+^ promoted S,S-(2,2′-diC_18:1_)-BMP isomerization better than alkaline earth metal ions such as Mg^2+^, Ca^2+^, and Ba^2+^ under neutral conditions using 100% S,S-(2,2′-diC_18:1_)-BMP liposomes (Fig. 6A). Lysosomes have functional roles in metal ion metabolism and homeostasis and in particular play a central role in iron metabolism (30, 31). The promotion of S,S-(2,2′-diC_18:1_)-BMP isomerization by Fe^2+^ or Fe^3+^ was dependent on the incubation time, the concentrations of these ions, and pH (Fig. 6B–E). The efficiency of the isomerization by Fe^3+^ was much better than that by Fe^2+^, which was comparable to that by Zn^2+^ (Fig. 5E). These effects on isomerization were suppressed in the presence of EDTA (Fig. 6D, E), confirming that these free ions act as promotors in the migration of acyl groups of S,S-(2,2′-diC_18:1_)-BMP. In addition, the molar ratio of S,S-(2,2′-diC_18:1_)-BMP/DODPC in liposomes affected the isomerization efficiency for both, Fe^3+^ and BSA (Fig. 6F). There are differences in the isomerization transition profiles of Fe^3+^ and BSA were not surprising in that ferric ions interact directly with BMP molecules through the negatively charged phosphates of BMP, whereas BSA interacts with lipid membranes that contain BMP.

BMPs are components of the inner membranes of late endosomes and lysosomes, and BMPs in some ILVs account for up to 70% of the total phospholipids in these vesicles (18). Therefore, it is possible that ILVs with a higher content of S,S-(2,2′-diC_18:1_)-BMP molecules are more sensitive to metal ions and proteins that promote S,S-(2,2′-diC_18:1_)-BMP isomerization than non-ILV lysosomal inner membranes when lysosomal pH is increased.

We also observed found that promotion of the isomerization of S,S-(2,2′-diC_18:1_)-BMP by BSA and Fe^3+^ under neutral conditions was suppressed in the presence of ATP (Supplemental Fig. S4). The inhibitory effect of ATP on the Fe^3+^-associated isomerization reaction (Supplemental Fig. S4B) was much greater than that by HSP70 (Fig. 5D, lanes 5 and 6) or BSA (Supplemental Fig. S4A, right panel). These proteins as well as ions are known to interact with nucleotides (50, 51, 52). Presumably, ATP could not only induce conformational changes in the tested proteins but also act as a chelator for the tested metal ions, resulting in an inhibitory effect.

Taken together, our in vitro findings suggest the existence of a complex and intricate regulatory system in cells for S,S-(2,2′-diacyl)-BMP isomerization, in which four factors, namely, proteins, metal ions, nucleotides, and pH, may be interrelated. These data suggest that pH is the most important factor for the acyl migration of S,S-(2,2′-diacyl)-BMP.

Finally, we explored whether the isomerization observed in vitro occur in living cells.

Using RAW-264.7 cells, we observed that the cells contain *sn*-1,3-specific lipase-sensitive BMP molecular species that were increased by increasing the luminal pH of late endosomes and lysosomes using CHL or NH_4_Cl (Fig. 7). Although CHL induced a significant increase in the LSF of cellular BMPs (Fig. 7A), the size of this LSF varied from experiment to experiment. However, it was repeatedly observed that the values of the LSF of BMP in CHL-treated cells were higher than those in CHL-untreated cells. Therefore, CHL may modulate not only cellular BMP content but also BMP molecular species. In addition, an increase of the LSF of BMPs by CHL was not always associated with increased phospholipid levels (Fig. 7B).

The absence of changes in total BMP was confirmed by RAW-264.7 cells treated with NH_4_Cl, a reagent that does not induce phospholipidosis (Fig. 7C, D). These results indicate that RAW-264.7 cells contain not only S,S-(2,2′-diacyl)-BMP but also other BMP isomers, such as S,S-(2,3′-diacyl)-BMP and (3,3′-diacyl)-BMP, as BMP components produced by the cell. This suggests that the cells have a capability to convert S,S-(2,2′-diacyl)-BMPs via S,S-(2,3′-diacyl)-BMPs to S,S-(3,3′-diacyl)-BMPs within the cells (Fig. 3E).

The in vitro studies demonstrated that proteins such BSA, HSA, and HSP70 and metal ions particularly, iron and zinc, promote the isomerization of S,S-(2,2′-diC_18:1_)-BMP via S,S-(2,3′-diC_18:1_)-BMP to S,S-(3,3′-diC_18:1_)-BMP under neutral conditions. Studies with RAW-264.7 cells demonstrated that lysosomes contain not only S,S-(2,2′-diacyl)-BMP but also S,S-(3,3′-diacyl)-BMPs, albeit at low levels at baseline. The fraction of S,S-(3,3′-diacyl)-BMP is increased by lysosomal alkalinization with CHL and NH_4_Cl.

**Fig. 8:**
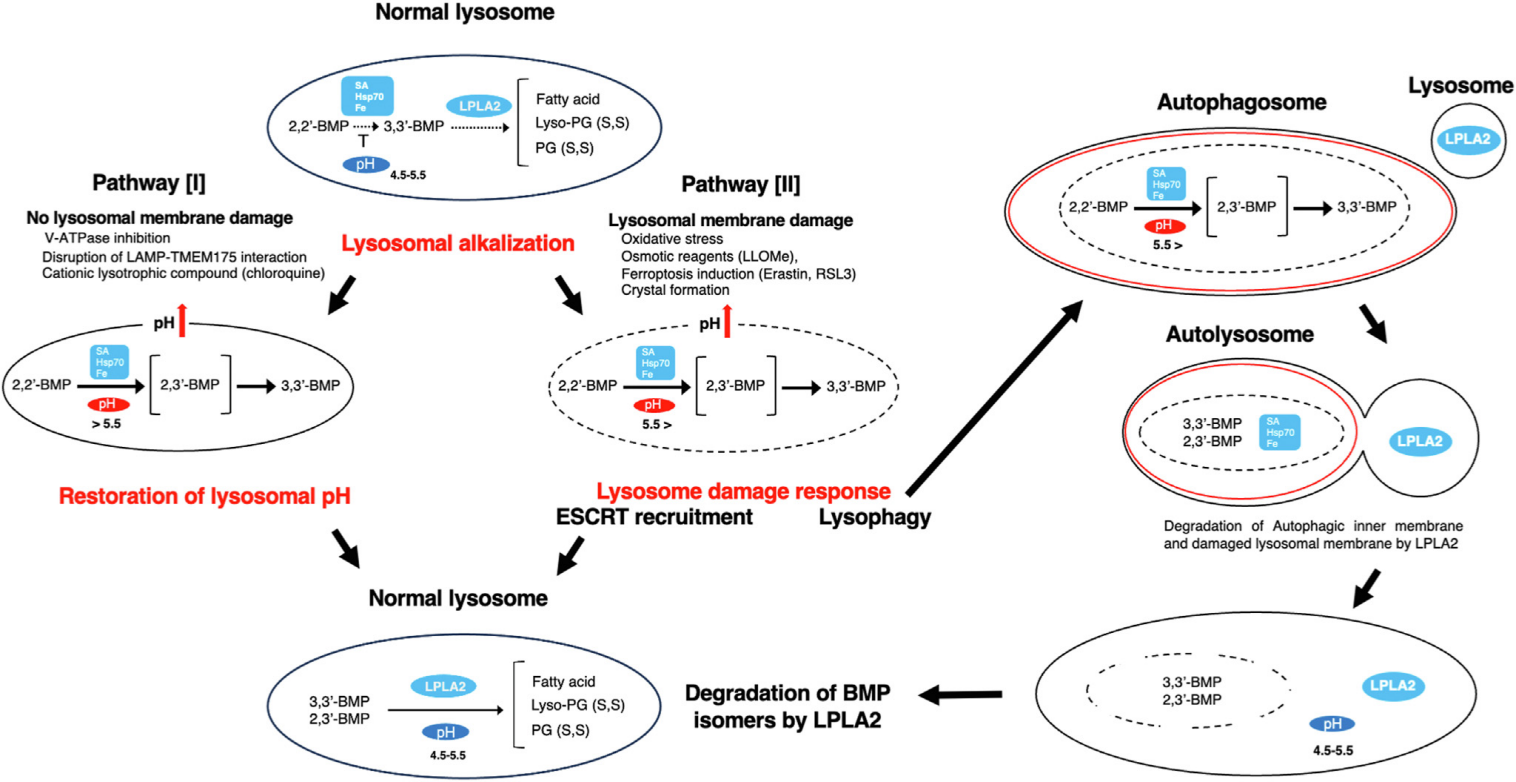
A model of isomerization and degradation of S,S-(2,2′-diacyl)-BMP in lysosomes. Proposed schematic of isomerization and degradation of S,S-(2,2′-diacyl)-BMP in lysosomes. The present results support pathway I. SA denotes serum albumin that is imported into lysosomes from the extracellular space via the endocytic pathway. HSP70, which is localized in lysosomes, is involved in lysosomal membrane stability. Furthermore, lysosomes play a central role in ferric and ferric iron metabolism. These proteins and metal ions do not act as isomerization promoters for S,S-(2,2′-diacyl)-BMP under the acidic conditions (pH 4.5–5.5), but rather through alkalinization of lysosomes (pH > 5.5). Lysosomal disorder caused by lysosomal alkalinization in membrane nonpermeabilized lysosomes can be overcome by reacidification. As a result, S,S-(2,3′-diacyl)-BMP and S,S-(3,3′-diacyl)-BMP generated by lysosomal alkalinization may be efficiently degraded by LPLA2 in normal lysosomes. An alternative alkalinization pathway (pathway II) may be induced via lysosomal membrane damage. Such permeabilized lysosomes can be repaired through the ESCORT machinery or targeted for lysophagy. BMP, bis(monoacylglycero)phosphate; LPLA2, lysosomal phospholipase A2.

To summarize, under normal conditions, S,S-(2,2′-diC_18:1_)-BMP is the predominant isomer present. Under conditions of stress such as lysosomal alkalinization, permeabilization, or oxidation factors that favor isomerization to S,S-(3,3′-diC_18:1_)-BMP may be present. Based on the pathobiology of such processes, a testable model of BMP isomerization coupled with catabolism by LPLA2 is proposed (Fig. 8). In this model, lysosomal alkalization, such as by V-ATPase inhibition, disruption of LAMP-TMEM175 interaction (53), or exposure to cationic lysosomotropic drugs such as CHL (54) (Fig. 8, pathway I), or increased membrane permeabilization of lysosomes, such as by induction of oxidative stress and ferroptosis (55, 56), exposure to ionophores such as LLOMe (57), or crystal formation (58) (Fig. 8, pathway II), could lead to an increase in the luminal pH and promote the conversion of S,S-(2,2′-diacyl)-BMP to S,S-(3,3′-diacyl)-BMP. In lysosomes with damaged membranes (Fig. 8, pathway II), iron ions and lysosomal proteins stored in the luminal space may be released into the cytoplasmic space. In such pathological states, permeabilized lysosomes are either targeted for lysophagy (59) or repaired by the ESCRT machinery (60). On the other hand, nonpermeabilized lysosomes with elevated pH (Fig. 8, pathway I) can be restored by reacidification of lysosomes. Reacidification may be achieved by increased intracellular cAMP (61) or by administration of biodegradable acidifying nanoparticles (62). Lysosomal repair may be associated with normal degradation by LPLA2 of S,S-(2,3′-diacyl)-BMP and S,S-(3,3′-diacyl)-BMP generated by exposure to ferric ion and lysosomal alkalinization.

## Data availability

All data is available in the main text and supplementary figures.

## Supplemental data

This article contains supplemental data.

## Conflict of interest

The authors declare that they have no conflicts of interest with the contents of this article.

## Figures and Tables

**Supplemental fig 4:**